# Prevalence of myopia: A large-scale population-based study among children and adolescents in weifang, china

**DOI:** 10.3389/fpubh.2022.924566

**Published:** 2022-07-25

**Authors:** Jie Zhang, Zhenhua Li, Jiantao Ren, Wenting Wang, Jiazhen Dai, Cong Li, Xudong Huang, Xianyong Sun, Lei Liu, Chunping Wang

**Affiliations:** ^1^Department of Environmental Hygiene, School of Public Health, Weifang Medical University, Weifang, China; ^2^Department of Ophthalmology, Weifang Eye Hospital, Weifang, China; ^3^Department of Ophthalmology, Guangdong Eye Institute, Guangdong Provincial People's Hospital, Guangdong Academy of Medical Sciences, Guangzhou, China; ^4^Department of Ophthalmology, Jincheng People's Hospital, Jincheng, China

**Keywords:** students, myopia, prevalence, cross-sectional studies, large scale

## Abstract

**Purpose:**

This study aimed to determine the prevalence of myopia among school-aged children and adolescents at the whole city level of Weifang, China.

**Methods:**

This study was a large scale school-based cross-sectional study among children and adolescents aged 5–20 years old. Participants were selected by the school-based registration system in 2020. All the subjects underwent spherical equivalent (SE) error with non-cycloplegic autorefraction measurement. Myopia was defined as an SE refraction of ≤-0.75 diopters (D) and graded into low myopia (−0.75 to −3.00 D), moderate myopia (−3.01 to −5.99 D), and high myopia (≤-6.00 D).

**Results:**

A total of 1,059,838 participants were eligible for this survey and 1,013,206 (95.6% participation rate) were selected through data quality control, which comprised 17 districts/counties and 1,254 schools, including 861 elementary schools, 313 middle schools, and 80 high schools. The mean age of participants was 11.57 ± 3.36 years (5–20 years), and the male-to-female ratio was 1.11. The whole city-level prevalence of total myopia was 75.35%. The prevalence of total myopia among the students in the Hanting District was 45.47%, but ≈82.37% of students living in Changyi have myopia. The overall prevalence of low myopia in elementary, middle, and high school students was 48.56, 47.30, and 31.62%, respectively, while high myopia (SE ≤ −6.00 D) prevalence was 1.12, 8.89, and 20.12%, respectively. The overall prevalence of myopia increased fastest in children aged 7–9 years old. The prevalence of high myopia was 7.59% for girls and 6.43% for boys, respectively (*p* < 0.001). The prevalence of myopia increased with increasing age and grade, but SE decreased with increasing age and grade.

**Conclusions:**

The current investigation demonstrated a high proportion of myopia among school students in the city of Weifang, and gradually increased with age, and the prevalence of myopia was the highest in Changyi areas. The high myopia prevalence for girls was higher than that in boys.

## Introduction

Myopia is one of the most common diseases, and is the single largest cause of visual impairment among children and adolescents globally (1). High myopia can result in serious pathologic events, such as myopic macular degeneration or retinal detachment, which lead to irreversible vision loss (2). It is estimated that about 1.4 billion people in the world suffered from myopia in 2000, and the number will reach 4.8 billion by 2050 (3). The potential global productivity loss associated with the burden of distance vision impairment caused by uncorrected myopia was estimated at US $244 billion in 2015 (4). Due to the influence of genetic and environmental factors, the incidence of myopia in adolescents and children around the world, especially in Asia, is still increasing rapidly (5).

Similarly, the prevalence of myopia in mainland China is also increasing year by year, which is higher than that in other East Asian countries (6, 7). A nationwide survey (2014) from China reported that ≈80% of students completing 12 years of school education are now myopic, and ≈10–20% of those people have high myopia (8). To date, there are many cross-sectional school-based studies on myopia among school-aged children in mainland China, while large-scale, covering the whole city level in this age group, there are relatively fewer, even though evaluating the estimates of myopia has a key role for policymakers to make appropriate decisions.

Weifang City is located in the area along the eastern coast of Shandong. As of 2021, the total population was 9,386,705, of which 17.37% of people were aged below 14 years old (https://www.sohu.com/a/471531178_99956894). Weifang has 17 districts/counties, and living habits, economic status, and medical and health resources are different in individualized districts or counties. Therefore, the distribution of myopia in adolescents and children may also be obviously different. The present large-scale study is aimed at evaluating the prevalence of myopia among primary and middle school students aged 5–20 years old in Weifang City.

## Methods

### Study design

This survey is a large-scale, population-based, cross-sectional epidemiology study undertaken to meet the ocular health requirements of school-aged children and adolescents for the entire city of Weifang from June to December 2020, within 16 districts/counties. This study was supported by the Weifang government. It conformed to the principles of the Declaration of Helsinki and has been approved by the Institutional Review Board of Weifang Eye Hospital. All participants provided their written informed consent form signed by their parents or guardians, as well as verbal assent from each participant.

### Population

The participants were school-age students from a school-based registration system, and studying at primary schools, middle schools, and high schools in each district or county of Weifang, Shandong Province, China.

### Data collection

Demographic data, such as age, gender, and education level were collected by teachers or guardians. All healthcare professionals were trained as investigators before the survey. To monitor the data's validity, a two-stage self-examination was performed. First, each school carried out the self-examination by simple random sampling of 5% of the individuals. Second, the manager of the study group randomly selected 5% of the children from 5% of the schools randomly selected for further verification. All children underwent a refractive examination with the same procedure, involving non-cycloplegic autorefraction (Topcon KR-800S AR) on both eyes. Before autorefractive testing, the autorefraction was calibrated by standard analog eyes and the cylindrical lens was adjusted to negative values. Each participant underwent autorefraction without cycloplegia three times in each eye, and the average value was adopted. Students wearing ordinary eyeglasses were instructed to remove them before the test was performed. A slit lamp examination was performed to assess cornea and lens status (cataract or clear lens, pseudophakic, and aphakic).

Individuals who underwent previous laser refractive surgery or cataract surgery, or used low-dose atropine, anti-myopia spectacles, anti-myopia multifocal soft contact lenses, and orthokeratology were excluded.

### Definition

The spherical equivalent (SE) error is calculated by adding the sum of the sphere power with half of the cylinder power. Myopia was defined as an SE refraction of ≤-0.75 diopters (D) and graded into low myopia (−0.75 to −3.00 D), moderate myopia (−3.01 to −5.99 D), and high myopia (≤-6.00 D) (9). Moreover, we determined the prevalence of high myopia where SE was ≤-5.0 D based on recent classification criteria (10).

### Statistical analyses

All statistical analyses were performed by SPSS v25.0 software (SPSS Inc., Chicago, IL, USA). Continuous data are described as mean ± standard division (SD). Categorical data are given as numbers (percentage). Prevalence rates with 95% confidence intervals (*CI*s) were described. Continuous data were compared between the groups using unpaired, two-tailed *t*-tests, and one-way analysis of variance (ANOVA) if normality assumptions were met, or using Wilcoxon's rank-sum test. Categorical data were compared between the groups using the chi-square test. The Pearson correlation coefficient was used to assess the correlation between the SE of the left and right eyes. As the SE of the two eyes were highly correlated (Pearson's correlation = 0.866, *p* < 0.05), and we used the SE of the right eye of the students as the basis for evaluating the development of myopia. The statistical tests were two-sided, and a *p*-value < 0.05 was considered statistically significant.

## Results

A total of 1,059,838 participants were eligible for this investigation and 1,013,206 (95.6% participation rate) completed all examinations, which comprised 17 districts/counties and 861, 313, and 80 elementary, middle, and high schools, respectively. There were 532,851 boys, accounting for 50.28% of the study participants. The mean age was 11.57 ± 3.36 (5–20) years.

**Figure 1:**
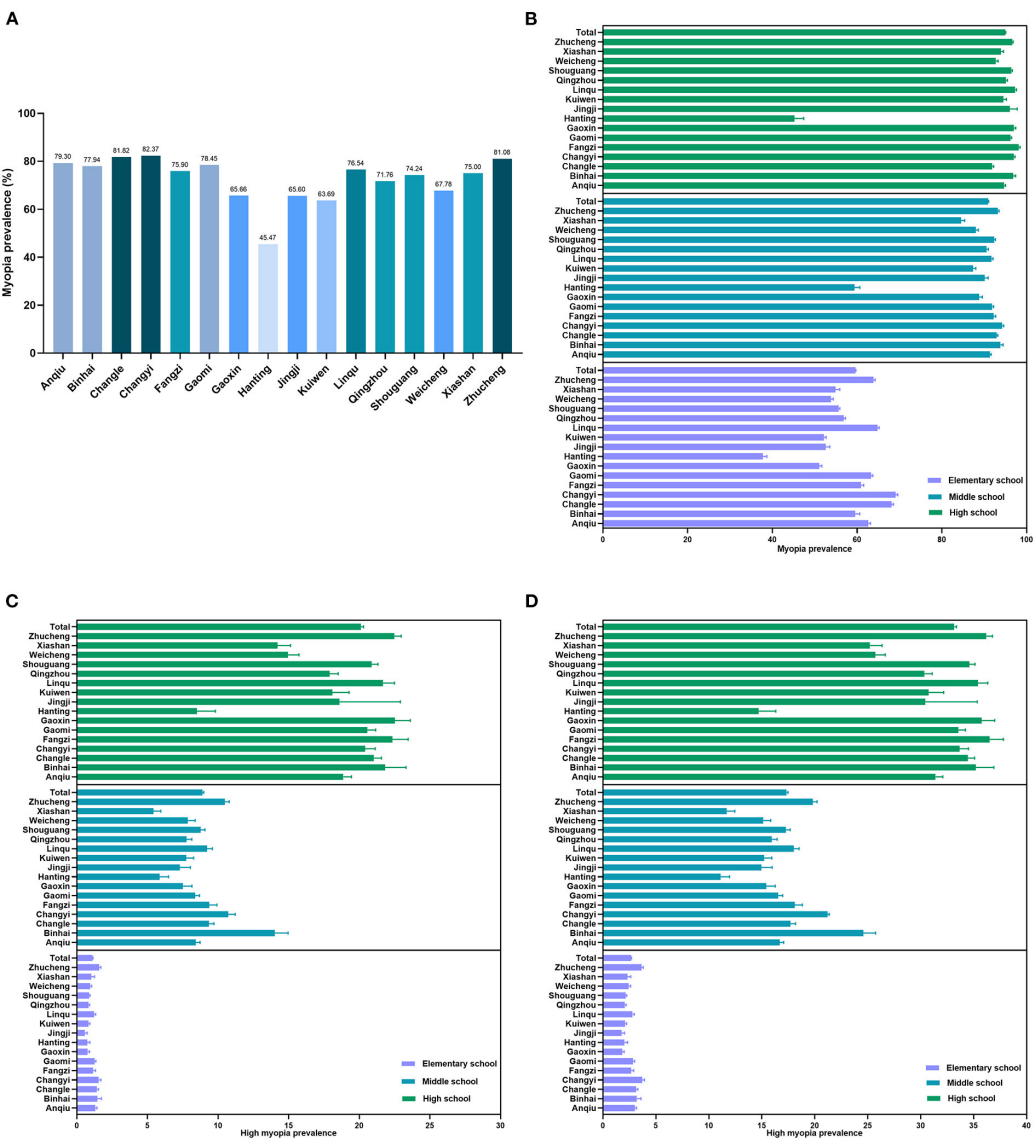
Myopia and high myopia prevalence of different regions in Weifang. **(A)** Myopia prevalence in 16 regions. **(B)** The myopia prevalence of different regions by school levels. **(C)** High myopia (spherical equivalent, SE ≤ −6.00 D) prevalence of different regions by school levels. **(D)** High myopia (SE ≤ −5.00 D) prevalence of different regions by school levels.

The overall prevalence of myopia was 75.35%, with mean SE was −2.14 ± 2.30 D (Table 1). The prevalence of total myopia among the students in the Hanting District was 45.47%, but ≈82.37% students living in the Changyi District have myopia (Figure 1A). Stratified by myopia grade, participants living in the Hanting District have the lowest prevalence of low myopia, moderate myopia, and high myopia (≤-5.00 D), while those in Jingji have the lowest prevalence of high myopia, which is defined as ≤-6.00 D (*p* < 0.001).

**Table 1:** Distribution of different grades of myopia in different regions (%).

**Location**	**Count (n)**	**SE, [mean (SD), D]**	**Myopia with SE** ≤ –**0.75 D (95%CI)**	**Low myopia with** −**3.00 D** ≤ **SE** ≤ –**0.75 D (95%CI)**	**Moderate myopia with** −**6.00 D**<**SE** < −**3.00 D(95%CI)**	**High myopia with SE** ≤ –**6.00D (95%CI)**	**Moderate myopia with** −**5.00 D**<**SE** < −**3.00 D (95%CI)**	**High myopia with SE** ≤ –**5.00 D (95%CI)**
Anqiu	95,447	–2.31 (2.26)	73.66 (46.03–46.67)	46.35 (46.03–46.67)	25.68 (25.40–25.96)	7.28 (7.11–7.44)	19.51 (19.25–19.76)	13.45 (13.23–13.67)
Binhai	17,077	–2.45 (2.41)	72.59 (71.92–73.26)	42.14 (41.40–42.89)	26.36 (25.70–27.02)	9.43 (9.00–9.88)	19.51 (18.91–20.11)	16.29 (15.73–16.85)
Changle	85,405	–2.55 (2.31)	77.06 (76.77–77.34)	45.42 (45.09–45.76)	27.47 (27.17–27.77)	8.92 (8.73–9.11)	20.69 (20.42–20.97)	15.67 (15.46–15.95)
Changyi	56,451	–2.51 (2.27)	77.05 (76.70–77.39)	46.07 (45.66–46.48)	28.01 (27.64–28.38)	8.29 (8.06–8.52)	21.05 (20.72–21.39)	15.25 (14.95–15.55)
Fangzi	37,544	–2.14 (2.28)	69.91 (69.44–70.37)	45.78 (45.28–46.29)	23.33 (22.90–23.76)	6.79 (6.53–7.05)	17.75 (17.37–18.14)	12.36 (12.03–12.70)
Gaomi	103,310	–2.24 (2.25)	72.45 (72.17–72.72)	47.55 (47.24–47.85)	23.79 (23.54–24.06)	7.11 (6.96–7.27)	17.99 (17.76–18.23)	12.91 (12.71–13.12)
Gaoxin	37,908	–1.68 (2.23)	58.61 (58.11–59.10)	42.72 (42.22–43.22)	17.44 (17.06–17.83)	5.49 (5.27–5.73)	13.14 (12.80–13.49)	9.79 (9.50–10.10)
Hanting	18,793	–0.65 (2.59)	41.21 (40.51–41.92)	29.12 (28.47–29.77)	13.14 (12.66–13.63)	3.21 (2.97–3.48)	10.05 (9.62–10.48)	6.31 (5.96–6.66)
Jingji	15,836	–1.48 (2.01)	57.94 (57.17–58.71)	46.00 (45.22–46.78)	16.46 (15.89–17.05)	3.14 (2.88–3.43)	12.95 (12.43–13.48)	6.66 (6.28–7.06)
Kuiwen	47,293	–1.46 (2.11)	56.70 (56.26–57.15)	43.74 (43.30–44.19)	16.05 (15.72–16.38)	3.89 (3.72–4.07)	12.40 (12.10–12.70)	7.54 (7.31–7.79)
Linqu	82,069	–2.04 (2.19)	69.95 (69.64–70.27)	49.18 (48.84–49.52)	21.27 (20.99–21.55)	6.09 (5.93–6.26)	16.10 (15.85–16.35)	11.26(11.04–11.48)
Qingzhou	89,234	–1.90 (2.17)	64.76 (64.44–65.07)	45.53 (45.21–45.86)	20.69 (20.42–20.95)	5.54 (5.39–5.69)	15.78 (15.54–16.02)	10.44 (10.24–10.65)
Shouguang	135,171	–2.18 (2.34)	68.47 (68.22–68.72)	42.78 (42.52–43.04)	23.98 (23.76–24.21)	7.47 (7.33–7.61)	18.01 (17.81–18.22)	13.44 (13.26–13.63)
Weicheng	50,204	–1.70 (2.17)	61.05 (60.62–61.48)	44.47 (44.03–44.90)	18.52 (18.18–18.86)	4.79 (4.60–4.98)	14.21 (13.90–14.51)	9.10 (8.85–9.36)
Xiashan	22,498	–2.06 (2.26)	69.42 (68.81–70.02)	45.25 (44.60–45.91)	23.81 (23.26–24.37)	5.94 (5.63–6.26)	18.31 (17.81–18.82)	11.44 (11.02–11.86)
Zhucheng	118,966	–2.55 (2.39)	75.82 (75.57–76.06)	43.75 (43.47–44.04)	27.95 (27.70–28.21)	9.37 (9.21–9.54)	20.80 (20.57–21.03)	16.52 (16.31–16.74)
Total	1,013,206	–2.14 (2.30)	69.41 (69.32–69.50)	44.96 (44.87–45.06)	23.41 (23.32–23.49)	6.98 (6.93–7.03)	17.68 (17.61–17.76)	12.70 (12.63–12.76)
P		<0.001		<0.001	<0.001	<0.001	<0.001	<0.001

*SE, spherical equivalent; D, diopters; CI, confidence interval*.

Table 2 shows the prevalence rate by age and gender. Girls have the lower SE compared with boys (*p* < 0.001). The prevalence of moderate myopia and high myopia was higher in girls than that in boys (*p* < 0.001). However, the prevalence of low myopia in girls was lower than that in boys (*p* < 0.001). The overall prevalence of low myopia was 44.96% (95% *CI*: 44.87–45.06%), and the 9–12 age groups had the proportion of more than 50%. The prevalence of high myopia (SE ≤ −6.00 D) was 6.98% (95% *CI*: 6.93–7.03%), and the 17–18 age groups had the highest prevalence more than 20%. However, the prevalence of high myopia (SE ≤ −5.00 D) was 12.70% (95% *CI*: 12.63–12.76%), and the 16–18 age groups had the highest prevalence of more than 30%.

**Table 2:** Distribution of different grades of myopia stratify by gender and age (%).

	**Count (n)**	**SE, [mean (SD), D]**	**Myopia with SE** ≤ –**0.75 D (95%CI)**	**Low myopia with** −**3.00 D** ≤ **SE** ≤ –**0.75 D (95%CI)**	**Moderate myopia with** −**6.00 D**<**SE** < −**3.00 D(95%CI)**	**High myopia with SE** ≤ –**6.00D (95%CI)**	**Moderate myopia with** −**5.00 D**<**SE** < −**3.00 D (95%CI)**	**High myopia with SE** ≤ –**5.00 D (95%CI)**
Gender
Male	532,851	–2.02 (2.26)	67.23 (67.10–67.35)	45.30 (45.17–45.44)	21.86 (21.75–21.97)	6.43 (6.36–6.49)	16.56 (16.47–16.66)	11.72 (11.64–11.81)
Female	480,355	–2.27 (2.33)	71.83 (71.70–71.96)	44.58 (44.44–44.72)	25.12 (25.00–25.24)	7.59 (7.52–7.67)	18.93 (18.82–19.04)	13.78 (13.69–13.88)
*P*		<0.001	<0.001	<0.001	<0.001	<0.001	<0.001	<0.001
Age (years)
≤ 7	144,999	–0.23 (1.22)	26.05 (25.82–26.28)	32.80 (32.56–33.05)	2.06 (1.99–2.14)	0.29 (0.27–0.32)	1.78 (1.71–1.85)	0.58 (0.54–0.62)
8	98,123	–0.69 (1.35)	42.66 (42.35–42.97)	47.85 (47.53–48.16)	4.48 (4.35–4.61)	0.38 (0.34–0.42)	3.95 (3.83–4.07)	0.91 (0.85–0.97)
9	86,275	–1.10 (1.52)	55.55 (55.22–55.88)	55.45 (55.12–55.78)	8.98 (8.79–9.17)	0.73 (0.67–0.78)	7.86 (7.68–8.04)	1.85 (1.76–1.94)
10	85,893	–1.51 (1.68)	66.10 (65.78–66.41)	57.99 (57.66–58.32)	14.65 (14.41–14.89)	1.45 (1.37–1.53)	12.50 (12.28–12.72)	3.60 (3.47–3.72)
11	78,596	–1.92 (1.82)	74.21 (73.90–74.52)	57.08 (56.73–57.43)	21.06 (20.77–21.35)	2.67 (2.56–2.78)	17.44 (17.18–17.71)	6.28 (6.12–6.46)
12	84,078	–2.35 (1.97)	80.81 (80.54–81.08)	54.18 (53.84–54.51)	27.20 (26.90–27.51)	4.78 (4.64–4.93)	21.58 (21.30–21.86)	10.41 (10.20–10.62)
13	102,419	–2.77 (2.08)	85.95 (85.73–86.16)	49.51 (49.20–49.81)	33.10 (32.81–33.39)	7.53 (7.37–7.69)	25.40 (25.14–25.67)	15.23 (15.01–15.45)
14	98,997	–3.15 (2.19)	89.13 (88.94–89.32)	44.16 (43.85–44.47)	37.46 (37.16–37.76)	10.71 (10.52–10.91)	27.76 (27.48–28.04)	20.41 (20.16–20.66)
15	85,404	–3.54 (2.27)	91.49 (91.30–91.68)	38.44 (38.11–38.77)	40.86 (40.53–41.19)	14.72 (14.48–14.96)	29.64 (29.34–29.95)	25.93 (25.64–26.23)
16	74,452	–3.84 (2.42)	92.47 (92.28–92.66)	33.25 (32.91–33.59)	42.26 (41.90–42.62)	18.91 (18.63–19.19)	29.90 (29.57–30.22)	31.27 (30.94–31.60)
17	49,658	–4.17 (2.42)	93.81 (93.59–94.02)	28.69 (28.29–29.09)	44.00 (43.57–44.44)	22.74 (22.37–23.11)	30.16 (29.76–30.57)	36.58 (36.16–37.01)
≥18	24,312	–4.23 (2.42)	93.98 (93.67–94.27)	27.71 (27.15–28.28)	44.45 (43.83–45.08)	23.29 (22.76–23.83)	30.01 (29.43–30.59)	37.73 (37.12–38.35)
*P*		<0.001	<0.001	<0.001	<0.001	<0.001	<0.001	<0.001

*SE, spherical equivalent; D, diopters; CI, confidence interval*.

Regarding the school level, the prevalence of low myopia was higher in the elementary school and middle school (with 48.56 and 47.3%, respectively). For high myopia (SE ≤ −6.00 D), high school had the highest prevalence (20.12%). Similarly, for high myopia (SE ≤ −5.00 D), high school also had the highest prevalence (33.17%) (Table 3). The prevalence of myopia in different regions by school level was shown in Figures 1B–D.

**Table 3:** Distribution of different grades of myopia stratify by school levels (%).

**Education phase**	**Grade**	**Count (n)**	**SE, [mean (SD), D]**	**Myopia with SE** ≤ –**0.75 D (95%CI)**	**Low myopia with** −**3.00 D** ≤ **SE** ≤ –**0.75 D (95%CI)**	**Moderate myopia with** −**6.00 D**<**SE** < −**3.00 D(95%CI)**	**High myopia with SE** ≤ –**6.00D (95%CI)**	**Moderate myopia with** −**5.00 D**<**SE** < −**3.00 D (95%CI)**	**High myopia with SE** ≤ –**5.00 D (95%CI)**
Elementary school	1st	93,683	–0.11 (1.20)	21.59 (21.32–21.85)	28.02 (27.73–28.30)	1.72 (1.64–1.81)	0.30 (0.26–0.33)	1.46 (1.39–1.54)	0.56 (0.51–0.61)
	2nd	92,363	–0.47 (1.28)	34.66 (34.35–34.96)	41.57 (41.25–41.89)	2.92 (2.81–3.03)	0.34 (0.31–0.38)	2.54 (2.44–2.65)	0.72 (0.67–0.78)
	3th	92,115	–0.84 (1.39)	47.97 (47.65–48.29)	52.06 (51.74–52.39)	5.63 (5.48–5.78)	0.45 (0.41–0.50)	5.00 (4.86–5.14)	1.08 (1.01–1.15)
	4th	88,433	–1.28 (1.56)	60.95 (60.62–61.27)	58.03 (57.71–58.36)	10.99 (10.79–11.20)	0.91 (0.85–0.98)	9.60 (9.41–9.80)	2.31 (2.21–2.41)
	5th	82,096	–1.67 (1.72)	69.86 (69.55–70.18)	58.40 (58.06–58.74)	17.16 (16.90–17.42)	1.71 (1.62–1.80)	14.44 (14.20–14.68)	4.42 (4.28–4.57)
	6th	80,242	–2.10 (1.87)	77.21 (76.92–77.50)	56.05 (55.71–56.40)	23.79 (23.50–24.09)	3.39 (3.26–3.51)	19.43 (19.16–19.71)	7.74 (7.56–7.93)
*P*			<0.001	<0.001	<0.001	<0.001	<0.001	<0.001	<0.001
	Total	528,932	–1.04 (1.65)	50.98 (50.84–51.11)	48.56 (48.42–48.69)	9.91 (9.82–9.99)	1.12 (1.09–1.15)	8.37 (8.29–8.44)	2.66 (2.62–2.70)
Middle school	7th	90,812	–2.52 (2.02)	83.17 (82.92–83.41)	52.55 (52.22–52.88)	29.68 (29.38–29.98)	5.85 (5.70–6.01)	23.29 (23.02–23.57)	12.24 (12.03–12.46)
	8th	103,301	–2.93 (2.12)	87.75 (87.55–87.95)	47.47 (47.16–47.77)	35.28 (34.99–35.57)	8.67 (8.50–8.85)	26.68 (26.41–26.95)	17.28 (17.05–17.51)
	9th	96,744	–3.30 (2.21)	90.25 (90.07–90.44)	42.19 (41.88–42.50)	38.99 (38.68–39.30)	11.96 (11.75–12.16)	28.74 (28.46–29.03)	22.21 (21.94–22.47)
*P*			<0.001	<0.001	<0.001	<0.001	<0.001	<0.001	<0.001
	Total	290,857	–2.93 (2.15)	87.15 (87.03–87.27)	47.30 (47.12–47.48)	34.77 (34.59–34.94)	8.89 (8.78–8.99)	26.31 (26.15–26.47)	17.34 (17.21–17.48)
High school	10th	77,265	–3.73 (2.28)	92.68 (92.50–92.87)	35.74 (35.40–36.08)	42.52 (42.17–42.87)	16.57 (16.31–16.83)	30.43 (30.10–30.75)	28.66 (28.35–28.99)
	11th	65,213	–3.92 (2.49)	92.29 (92.08–92.49)	31.23 (30.87–31.59)	42.55 (42.17–42.93)	20.44 (20.13–20.75)	29.78 (29.43–30.14)	33.21 (32.84–33.57)
	12th	50,939	–4.39 (2.39)	94.95 (94.75–95.14)	25.86 (25.48–26.25)	45.21 (44.78–45.64)	25.12 (24.74–25.50)	30.36 (29.96–30.76)	39.97 (39.54–40.39)
*P*			<0.001	<0.001	<0.001	<0.001	<0.001	<0.001	<0.001
	Total	193,417	–3.97 (2.39)	93.14 (93.03–93.26)	31.62 (31.41–31.82)	43.24 (43.02–43.46)	20.12 (19.95–20.30)	30.19 (29.99–30.40)	33.17 (32.96–33.38)

*SE, spherical equivalent; D, diopters; CI, confidence interval*.

**Figure 2:**
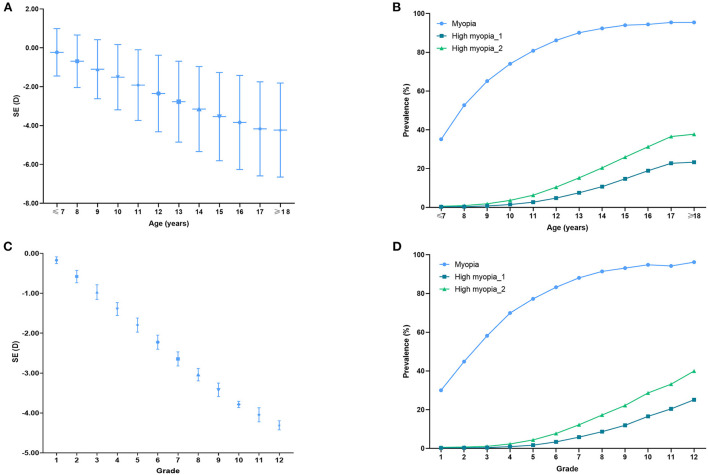
Spherical equivalent (SE) distribution and myopia prevalence of different ages and grades. **(A)** SE distribution for school students by age. **(B)** Myopia prevalence for school students by age. **(C)** SE distribution for school students of different grades. **(D)** Myopia prevalence for school students of different grades. High myopia_1: SE ≤ −6.00 D; High myopia_2: SE ≤ −5.00 D.

Figure 2 shows mean SEs for each age and grade (A and C) and the prevalence of myopia by age and grade (B and D). Participants aged over 18 years old and studying in the 12th grade had the lowest SE. Consistently, those participants aged over 18 years old and studying in their 12th grade had the highest prevalence of total and high myopia.

## Discussion

This large-scale, school-based, cross-sectional, city-wide study evaluated the prevalence and distribution of myopia in Chinese children and adolescents in Weifang. This study offers three key findings: (1) the whole city levels' prevalence of total myopia was 75.35% among 1,013,206 children and adolescents (5–20-year old) living predominantly in urban and suburban localities of Weifang, Shandong Province, China; (2) girls had a lower prevalence of low myopia but higher moderate and high myopia proportions; (3) participants in the elementary and middle school had higher low myopia proportions, while participants in the high school had a higher prevalence of high myopia. Current study provides key preliminary outcomes for the improvement of myopia control program and extension to other city-levels of the country.

To date, myopia prevention and control have become a national strategy in China. The vision of young people is related to the future of the country and the nation, and the whole society must attach great importance to prevent diseases. Previously, there were 19 population-based studies using cycloplegia refraction in China. Dong et al. revealed that the pooled prevalence of myopia was 32.96% (95% *CI*: 22.13–43.79%) aging 3–18 years and speculated that the myopia prevalence in 2050 among Chinese children and adolescents aged 3–19 years will be ≈84% (11). However, in our survey, the prevalence of total myopia was ranging from 45.47 to 82.37% among adolescents aged 5–20 years, which was higher than that in Dong et al. reports. This difference may be due to the different cycloplegia refraction between our and their studies. Moreover, we estimate that the actual situation of myopia prevalence by 2050 may be more serious than those estimates. In a recent population-based screening pipeline, which involved a million scale children and adolescents in Wenzhou, myopia and high myopia were defined as SE ≤ −1.00 D and SE ≤ −6.00 D, respectively (12). To compare with the Wenzhou study outcomes, we performed a further analysis based on their criteria. The overall prevalence of myopia (Supplementary Table 1) was 63.91% (95% *CI*: 63.81–64.00%), which was higher than that among the 6–20-year-old participants in Wenzhou (55.83%, 95% *CI*: 55.73–55.93%). Similarly, our further analysis found that the prevalence of myopia and high myopia in different genders, ages Supplementary Table 2), and educational levels (Supplementary Table 3) in Weifang were higher than those reports in Wenzhou. However, the underlying reason for these differences are still unknown, which may be related to potential factors, such as lifestyle, study burden, or genetic background. There is a need to perform more city-level studies of myopia to shed light on evidence for prevention.

In our study, it was found that the prevalence of myopia in the Hanting District was relatively lower. Hanting District is an economic development zone in the Weifang City. Children from families with lower household income and lower maternal education became more often myopic than those from socioeconomically advantaged families (13). The population living in the Hanting District is dominated by young people, with high per capita income and education levels. This part of the population pays more attention to the prevention and control of children's myopia, which may be the reason for the low prevalence of myopia. Notably, participants living in Changle County, Changyi County-city, and Zhucheng County-city had more than 80% proportions of myopia. Therefore, the prevention of myopia in urban-rural fringes and rural areas is also very serious and worthy of attention (14).

Another school-based cross-sectional study enrolled 3–14-year-old Chinese children in Chengdu by Wang et al. demonstrated that the prevalence of low myopia in the girls was higher than that in the boys (28.4 vs. 25.0%), while the prevalence of moderate myopia (9.5 vs. 10.1%), and high myopia (SE ≤ −6.00 D, 1.7 vs. 1.7%) did not significantly differ between the girls and boys (15). In our study, the total prevalence of myopia in the girls was higher than that in the boys, which is consistent with those in previous studies (16, 17). In contrast, in our study, girls presented low myopia prevalence than boys, but the proportion of moderate myopia and high myopia was higher in girls than in boys. That is to say, the total prevalence of myopia in the girls was higher than that in the boys, and this difference was mainly determined by the cases of high myopia in Weifang. Our finding was consistent to Li et al., who revealed that the prevalence rates of moderate and high myopia were higher in girls than in boys, the opposite to Wang et al (15). The discrepancy may be that the age of children and adolescents in our cross-sectional study were ranging from 5 to 20 years old, but Wang et al. included children aged 3–14 years and defined myopia as SE ≤ −0.50 D.

Regarding myopia severity, high myopia can cause vision-threatening complications, such as rhegmatogenous retinal detachment (RRD), choroid neovascularization (CNV), and macular hemorrhage (MH), which harms public visual health (18, 19). Therefore, preventing and controlling the occurrence and progression of high myopia is a critical public health issue. In our observations, the presence of high myopia is associated with increasing age and SE drifts to be more myopic, which is consistent with previous studies (6, 20). Simultaneously, the prevalence of high myopia is particularly prominent in high school students, while primary and middle school students are more likely to have low and moderate myopia. Therefore, this suggests that we should focus on the prevention and control of high myopia for high school students to avoid causing complications. Nevertheless, the primary and middle school students should be paid more attention to the control of low and moderate myopia and avoid developing into high myopia.

The strengths of our study included a large sample size, multi-dimensional features, and a whole city scale observation. However, there are limitations to the present study. First, cycloplegia refraction was not adopted in the current study due to the large scale investigation, and this non-cycloplegia refraction would overestimate the myopia prevalence of children. However, our findings provided some basis for future studies. Second, our study is a cross-sectional study, which would reduce the quality of evidence to a certain extent. Third, although this is a large scale study with high response rate (95.6%), this was a school-based investigation rather than a population-based cohort. Thus, it might be considered that the overall prevalence rates of myopia may be biased because some children who did not go to school or lived in special education school (i.e., mentally handicapped or orphans living in Children's Welfare Institute) were not included, which may have contributed to impact on myopia prevalence. Finally, students after previous special myopia treatment do not reflect the real myopia situation. Herein, those students who underwent previous laser refractive surgery or used low-dose atropine, anti-myopia spectacles, anti-myopia multifocal soft contact lenses, and orthokeratology were excluded in current study. Considering that, the mean refraction in our study would be underestimated. Further well designed, longitudinal studies covering the completeness of whole children and adolescents should be conducted in future.

## Conclusion

There is a strikingly high prevalence of total myopia in children and adolescents aged 6–20 years old in Weifang. Students living in the Hanting economic development zone had the lowest prevalence of myopia. The prevalence of myopia in girls is higher than that in boys, particularly in high myopia. With increasing age, the prevalence of both total myopia and high myopia increases, and SE drifts to be more myopic. The high myopia rate in high school students is higher, so it is necessary to take measures to prevent the progression of myopia.

## Data availability statement

The original contributions presented in the study are included in the article/supplementary material, further inquiries can be directed to the corresponding author/s.

## Ethics statement

The studies involving human participants were reviewed and approved by Weifang Eye Hospital. Written informed consent to participate in this study was provided by the participants' legal guardian/next of kin.

## Author contributions

All authors listed have made a substantial, direct, and intellectual contribution to the work and approved it for publication.

## Conflict of interest

The authors declare that the research was conducted in the absence of any commercial or financial relationships that could be construed as a potential conflict of interest.

## Publisher's note

All claims expressed in this article are solely those of the authors and do not necessarily represent those of their affiliated organizations, or those of the publisher, the editors and the reviewers. Any product that may be evaluated in this article, or claim that may be made by its manufacturer, is not guaranteed or endorsed by the publisher.
