# Supplementary material for: Prevalence of myopia: A large-scale population-based study among children and adolescents in weifang, china
Source: Front Public Health. 2022 Jul 25;10:924566. doi: 10.3389/fpubh.2022.924566 (PMC9358211; doi:10.3389/fpubh.2022.924566)
Supplement: Supplementary file 1 [file Table_1.pdf]

Supplementary Table 1. Distribution of different grades of myopia in different regions according to Wenzhou standard (%)

**Location**	**Count (n)**	**Myopia with SE ≤ -1.00 D (95%CI)**	**Low myopia with -3.00 D < SE ≤ -1.00 D (95%CI)**	**Moderate myopia with -6.00 D < SE ≤ -3.00 D (95%CI)**	**High myopia with SE ≤ -6.00 D (95%CI)**
Anqiu	95,447	68.39 (68.09-68.68)	33.75 (33.45-34.05)	27.36 (27.08-27.64)	7.28 (7.11-7.44)
Binhai	17,077	67.57 (66.86-68.27)	30.66 (29.97-31.36)	27.48 (26.81-28.15)	9.43 (9.00-9.88)
Changle	85,405	72.26 (71.96-72.56)	34.11 (33.79-34.43)	29.23 (28.92-29.54)	8.92 (8.73-9.11)
Changyi	56,451	71.71 (71.34-72.08)	33.57 (33.18-33.96)	29.85 (29.47-30.23)	8.29 (8.06-8.52)
Fangzi	37,544	64.35 (63.86-64.84)	32.72 (32.25-33.20)	24.84 (24.40-25.28)	6.79 (6.53-7.05)
Gaomi	103,310	66.55 (66.26-66.84)	34.02 (33.73-34.31)	25.42 (25.15-25.68)	7.11 (6.96-7.27)
Gaoxin	37,908	52.95 (52.44-53.45)	28.76 (28.31-29.22)	18.69 (18.30-19.09)	5.49 (5.27-5.73)
Hanting	18,793	37.53 (36.84-38.23)	20.34 (19.76-20.92)	13.98 (13.49-14.48)	3.21 (2.97-3.48)
Jingji	15,836	51.22 (50.44-52.00)	30.49 (29.78-31.22)	17.58 (16.99-18.18)	3.14 (2.88-3.43)
Kuiwen	47,293	50.38 (49.93-50.83)	29.27 (28.87-29.69)	17.22 (16.88-17.56)	3.89 (3.72-4.07)
Linqu	82,069	63.41 (63.08-63.74)	34.43 (34.11-34.76)	22.88 (22.60-23.17)	6.09 (5.93-6.26)
Qingzhou	89,234	58.58 (58.25-58.90)	30.98 (30.68-31.28)	22.06 (21.79-22.33)	5.54 (5.39-5.69)
Shouguang	135,171	63.35 (63.10-63.61)	30.42 (30.17-30.66)	25.46 (25.23-25.70)	7.47 (7.33-7.61)
Weicheng	50,204	55.16 (54.72-55.59)	30.78 (30.38-31.19)	19.58 (19.24-19.93)	4.79 (4.60-4.98)
Xiashan	22,498	64.05 (63.42-64.68)	32.81 (32.20-33.43)	25.30 (24.74-25.88)	5.94 (5.63-6.26)
Zhucheng	118,966	71.00 (70.74-71.26)	32.01 (31.75-32.28)	29.61 (29.35-29.87)	9.37 (9.21-9.54)
Total	1,013,206	63.91 (63.81-64.00)	32.01 (31.92-32.10)	24.92 (24.83-25.00)	6.98 (6.93-7.03)
P		<0.001	<0.001	<0.001	<0.001

SE: spherical equivalent; D: diopters; CI: confidence interval.

Supplementary Table 2. Distribution of different grades of myopia stratify by gender and age according to Wenzhou standard (%)

	Count (n)	Myopia with SE ≤ -1.00 D (95%CI)	Low myopia with -3.00 D < SE ≤ -1.00 D (95%CI)	Moderate myopia with -6.00 D < SE ≤ -3.00 D (95%CI)	High myopia with SE ≤ -6.00 D (95%CI)
Gender					
Male	532,851	61.44 (61.31-61.57)	31.73 (31.61-31.86)	23.28 (23.16-23.39)	6.43 (6.36-6.49)
Female	480,355	66.64 (66.51-66.78)	32.31 (32.18-32.44)	26.74 (26.62-26.87)	7.59 (7.52-7.67)
P		<0.001	<0.001	<0.001	<0.001
Age (years)					
≤7	144,999	19.43 (19.23-19.64)	16.83 (16.64-17.03)	2.31 (2.23-2.39)	0.29 (0.27-0.32)
8	98,123	34.22 (33.92-34.51)	28.78 (28.50-29.07)	5.05 (4.92-5.19)	0.38 (0.34-0.42)
9	86,275	47.05 (46.72-47.38)	36.29 (35.97-36.61)	10.03 (9.83-10.24)	0.73 (0.67-0.78)
10	85,893	58.30 (57.97-58.63)	40.73 (40.40-41.06)	16.12 (15.88-16.37)	1.45 (1.37-1.53)
11	78,596	67.58 (67.26-67.91)	42.03 (41.68-42.38)	22.88 (22.59-23.18)	2.67 (2.56-2.78)
12	84,078	75.10 (74.81-75.39)	41.06 (40.72-41.39)	29.26 (28.95-29.57)	4.78 (4.64-4.93)
13	102,419	81.30 (81.06-81.54)	38.60 (38.30-38.90)	35.18 (34.88-35.47)	7.53 (7.37-7.69)
14	98,997	85.48 (85.26-85.70)	35.00 (34.71-35.30)	39.76 (39.45-40.07)	10.71 (10.52-10.91)
15	85,404	88.69 (88.48-88.91)	31.03 (30.72-31.34)	42.95 (42.62-43.28)	14.72 (14.48-14.96)
16	74,452	90.11 (89.89-90.32)	26.88 (26.57-27.20)	44.32 (43.96-44.67)	18.91 (18.63-19.19)
17	49,658	91.99 (91.74-92.22)	23.45 (23.08-23.83)	45.81 (45.37-46.25)	22.74 (22.37-23.11)
≥18	24,312	91.12 (91.78-92.46)	22.47 (21.94-23.00)	46.36 (45.74-46.99)	23.29 (22.76-23.83)
P		<0.001	<0.001	<0.001	<0.001

SE: spherical equivalent; D: diopters; CI: confidence interval.

Supplementary Table 3. Distribution of different grades of myopia stratify by school levels according to Wenzhou standard (%)

Education phase	Grade	Count (n)	Myopia with SE ≤ -1.00 D (95%CI)	Low myopia with -3.00 D < SE ≤ -1.00 D (95%CI)	Moderate myopia with -6.00 D < SE ≤ -3.00 D (95%CI)	High myopia with SE ≤ -6.00 D (95%CI)
Elementary school	1st	93,683	15.73 (15.50-15.96)	13.52 (13.31-13.75)	1.90 (1.81-1.99)	0.30 (0.26-0.33)
	2nd	92,363	26.69 (26.41-26.98)	23.06 (22.79-23.33)	3.29 (3.18-3.41)	0.34 (0.31-0.38)
	3th	92,115	31.51 (32.21-32.81)	32.51 (32.21-32.81)	6.34 (6.19-6.51)	0.45 (0.41-0.50)
	4th	88,433	52.45 (52.12-52.78)	39.27 (38.95-39.59)	12.27 (12.06-12.49)	0.91 (0.85-0.98)
	5th	82,096	62.57 (62.24-62.90)	42.05 (41.71-42.38)	18.82 (18.55-19.09)	1.71 (1.62-1.80)
	6th	80,242	70.98 (70.67-71.30)	41.85 (41.51-42.19)	25.75 (25.44-26.05)	3.39 (3.26-3.51)
P			<0.001	<0.001	<0.001	<0.001
	Total	528,932	43.54 (43.41-43.68)	31.52 (31.40-31.65)	10.89 (10.81-10.98)	1.12 (1.09-1.15)
Middle school	7th	90,812	77.89 (77.62-78.16)	40.31 (39.99-40.63)	31.72 (31.42-32.03)	5.85 (5.70-6.01)
	8th	103,301	83.48 (83.26-83.71)	37.37 (37.07-37.67)	37.44 (37.15-37.74)	8.67 (8.50-8.85)
	9th	96,744	87.03 (86.82-87.25)	33.80 (33.51-34.10)	41.27 (40.96-41.58)	11.96 (11.75-12.16)
P			<0.001	<0.001	<0.001	<0.001
	Total	290,857	82.92 (82.78-83.05)	37.10 (36.93-37.28)	36.93 (36.76-37.11)	8.89 (8.78-8.99)
High school	10th	77,265	90.20 (89.99-90.41)	28.99 (28.67-29.32)	44.64 (44.29-44.99)	16.57 (16.31-16.83)
	11th	65,213	90.09 (89.86-90.32)	25.21 (24.88-25.55)	44.44 (44.06-44.82)	20.44 (20.13-20.75)
	12th	50,939	93.37 (93.15-93.58)	21.21 (20.85-21.56)	47.05 (46.61-47.48)	25.12 (24.74-25.50)
P			<0.001	<0.001	<0.001	<0.001
	Total	193,417	91.00 (90.87-91.13)	25.67 (25.47-25.86)	45.21 (44.98-45.43)	20.12 (19.95-20.30)

SE: spherical equivalent; D: diopters; CI: confidence interval.
